# Carbon Footprint of Laparoscopic Appendicectomy: A Prospective Observational Study at a Tertiary Care NHS Trust Hospital

**DOI:** 10.7759/cureus.84870

**Published:** 2025-05-27

**Authors:** George M Khalil, Nida Khan, Jaciron Kamalathasan, Muhammad Qasim, Reem Ahmed, Muhammad S Sajid

**Affiliations:** 1 Department of Digestive Disease and General Surgery, Royal Sussex County Hospital, Brighton, GBR; 2 Department of General Surgery, Brighton and Sussex Medical School, Brighton, GBR

**Keywords:** appendicitis, carbon dioxide emissions, carbon footprint, environmental sustainability, laparoscopic appendicectomy, laparoscopic surgery (lp), s: climate change

## Abstract

Introduction

Climate change is a significant global challenge as a result of rising greenhouse gas emissions. The National Health Service (NHS), as one of the biggest contributors to the United Kingdom's emissions, has a net-zero carbon emissions target. Operating theatres have a large resource burden in hospitals, and the laparoscopic appendicectomy (LA) is one of the most commonly performed procedures. As of yet, the laparoscopic appendicectomy has not been effectively carbon footprinted.

Aims

The objective of this study is to calculate the carbon footprint of the laparoscopic appendicectomy (LA) in grams of carbon dioxide (CO2) produced that results in an environmental impact. In addition, we also identify the biggest contributors to the carbon footprint and create a set of recommendations for CO2 emission reduction.

Methods

Carbon footprint-related data was collected for using surgical equipment, electricity utilisation and consumption of CO2 used for pneumoperitoneum, over 23 consecutive LAs. Peer-reviewed literature was used to determine the footprint of the equipment via a lifecycle assessment. The average carbon emissions for pneumoperitoneum, theatre overheads (mainly heating, ventilation and air conditioning (HVAC)) and electrical equipment were calculated.

Results

The average CO2 emission for an appendicectomy was 28258±1256 (24381-36267) grams. Variation was largely secondary to prolonged operations (Spearman's rank correlation coefficient = 0.95) due to technical difficulties. Major contributors in carbon footprinting were pre-set (reusable items) (23%), pre-set (disposable items) (40%), theatre overheads (HVAC and lights) (16%), CO2 pneumoperitoneum (9%), scrubs and personal protective equipment (PPE) (6%), additional equipment (5%) and utilisation of electrical equipment (1%).

Conclusion

The LA is a carbon-intensive procedure. CO2 emissions from LAs may be reduced by switching over to reusable equipment and increasing operating room efficiency. The green theatre checklist provides excellent guidelines on this. More research is needed into less environmentally impactful gases to achieve a pneumoperitoneum. This study provides data to guide targeted interventions and quantify the degree of offsetting needed to achieve net-zero emissions for laparoscopic surgery.

## Introduction

The environmental burden of carbon dioxide (CO2) emissions is significant; it is estimated that for every 4,434 metric tons of CO2 released, one premature death occurs globally [1]. Climate change is the biggest global health threat of the 21st century, as stated in a joint commission held by University College London Institute for Global Health and The Lancet [2]. This disproportionately affects the poorest individuals. Climate change results in a rise in Earth temperatures, the spread of vector-borne disease, flooding, air pollution and drought [3]. As we change our climate, we also increase the occurrence of respiratory disorders, infectious diseases and global disasters. Historically, medical advancements have improved physical health, but the resulting environmental burden may now contribute to global disease risk [4].

The greenhouse effect is a well-understood phenomenon that can be observed as the cause of the Earth's warmth. Greenhouse gases absorb heat from the sun and hence trap this heat in the Earth's atmosphere, creating the so-called 'greenhouse effect'. This is necessary for the Earth to maintain a temperature that can support life; however, this same phenomenon also accounts for the rise in world temperatures. Since the Industrial Revolution in 1750, the atmospheric concentrations of methane [5] and carbon dioxide [6] have increased by around 150% and 50%, respectively. This, in turn, explains a rise in the global average temperature by around 1°C, projected to be around 1.5°C by 2050 [7].

The National Health Service (NHS) is the biggest employer in the United Kingdom and the world's largest publicly funded healthcare system. With some 17 million inpatient admissions every year and around 270 million primary care appointments, the NHS produces around 5% of the United Kingdom's greenhouse gas emissions. In 2019 alone, NHS England produced 25 megatons of CO2 equivalent. As an institution that endeavours to improve the health of, and care for, its population, the NHS has a duty to reduce its contribution to global warming, if not only for the health of its patients [8]. Having noticed this, the NHS in all countries of the United Kingdom has made a commitment to net zero by 2040, with a target of an 80% reduction in emissions by 2032. As a result of this, an accurate estimate of the emission productions of all its components is required, and shared ownership of this goal needs to be achieved by all its constituents [9].

Operating theatres are known to be the greatest consumers of energy in hospitals, using around 3-6 times the energy compared to other parts of the hospital. Most of this energy is spent maintaining ventilation and temperature [10]. Around 59% of NHS CO2 emissions are related to the supply chain, of which the largest contributor is medical equipment. It is therefore not a surprise that operating theatres have a large resource burden and are hence a major contributor to the carbon footprint in the NHS [11]. Carbon footprinting is a process that calculates the output of greenhouse emissions equivalent to a weight of CO2 by certain products or procedures. The carbon footprint is useful to calculate as it allows identification of the processes and products that result in the largest production of greenhouse gases. It also allows us to quantify the effectiveness of our interventions to mitigate this [11]. Finally, a complete reduction in greenhouse gas emissions is impossible, but by understanding the emissions of the NHS's components, we can better plan for how to offset them.

Appendicitis is one of the most common surgical ailments, occurring at an incidence of around one in 1,000 persons per year [12]. There are around 50,000 appendicectomies performed in the United Kingdom every year [13], making it the most performed emergency surgical procedure [14] and one of the most common procedures generally. Thus far, although thought has been put into identifying how to reduce the carbon footprint of appendicectomies, the actual footprint of the procedure has not been calculated. As one of the most common procedures, its cumulative footprint will be significant, notwithstanding its importance as a benchmark for other laparoscopic procedures. Many other procedures have been footprinted in the literature, including cataract surgery [15], laparoscopic hemicolectomies [16] and even an entire renal service [17]. Rizan et al. describe 12 other procedures in their review of carbon footprinted operations [11], each using slightly different methods and with their own limitations.

Multiple actions have been proposed to reduce the footprint of minimally invasive surgery, with much attention paid to reducing the use of disposable equipment [18]. Minimally invasive surgery has been shown to produce good outcomes compared to its open counterpart in many procedures and is the gold standard method of most appendicectomies. However, this comes at a cost to the environment as it is an energy-intensive method that requires specialist equipment [19]. Extensive thought has been put into the carbon footprint of surgical equipment [20] and where the sets can be made more carbon-efficient [18]. The Green Surgery Report, published by the UK Health Alliance on Climate Change in collaboration with Brighton and Sussex Medical School, Centre for Sustainable Healthcare, outlines an extensive number of peer-reviewed interventions that can reduce the carbon footprint of surgery [21]. In this prospective observational study, we aim to measure the carbon footprint of the laparoscopic appendicectomy (LA) and, through this, hope to identify processes that could be improved by the implementation of recommendations from the Green Surgery Report.

## Materials and methods

The carbon footprint of the LA was calculated by incorporating all possible variables that are responsible for carbon emissions. This included four major domains: equipment (reusable and non-reusable), CO2 insufflation needed to create a pneumoperitoneum, electrical equipment and theatre overheads. Within a single tertiary care centre, data on the equipment and energy used were collected for 23 consecutive LAs. Appendicectomies were excluded if they were open, converted to open or aborted.

Equipment

The equipment footprint was referenced from Rizan et al [20]. In their study, Rizan et al. used a process-based life cycle approach to calculate the footprint of an inventory of individual products used for LAs, data for which was collected at the same trust as this study. The supplemental material of Rizan et al.'s study noted the footprint of equipment under different waste streams. The footprint of all the equipment used for every appendicectomy was calculated and averaged. The equipment was broken down into reusable 'set' items, non-reusable 'set' items and non-reusable 'variable’ items. Data on the number of people who scrubbed into the operation was collected, and estimates were made for the personal protective equipment (PPE) used. It was assumed that all those scrubbed had double-gloved and used hospital-provided scrubs. A list of all the equipment for which data was collected is given in Table 1. At times, there was no reference available for the supplemental specialist equipment used; this was limited to echelon laparoscopic staplers (used on two occasions) or clips (used also on two occasions), Veress needles (used on one occasion) and Robinson drains (used on six occasions). This resulted in the exclusion of these items from the calculation. The data collection sheet used by the surgical team to note down equipment usage is shown in the Appendices. All data was then transferred into an Excel spreadsheet for calculation (Microsoft Corp., Redmond, WA). All the equipment used, for which data was collected, was standardised at our trust; this was determined by discussion with the theatre scrub staff and runners who would set up the equipment for the LAs, the exception to this being the additional equipment mentioned earlier, which was not included in the calculation. There were no multiple variations of different equipment, such as ports, nor were there options for reusable or non-reusable versions. The uniformity in our trust is aided by the fact that most equipment comes in pre-packed sets.

**Table 1:** Equipment for which usage data was collected

Equipment	
ChloraPrep	Basic surgical set
Quiver and clip	Laparoscopic set
Laparoscope cover	Laparoscope
6 patient drapes/patient (single use)	Scrubs
Absorbent towel pack	Gowns
Blade	Facemasks
Sharps box	Gloves
Light handles	Surgical hat
Small swab packs (5)	Endoloops (assumed as sutures)
Insufflation tubing	Sutures
Antifog	Local anaesthetic
12 mm port	Syringes (20 mL/10 mL)
5 mm ports dual pack	1 L saline wash
Suction	Endoscopic clip applicator
Laparoscopic scissors	Tonsil swabs
Bert bag	
Needle	
Histology pot	
Suction receptacle	
Needle counter	
Camera cover (used also for insufflation tubing)	

Pneumoperitoneum

For each LA, data were collected on the length of the surgery from the time of the entry of the patient into the operating room to incision, and from incision to the skin closure. The average litres per minute of CO2 used in surgical time from incision to closure was calculated from the data collected on CO2 use for seven appendicectomies. This was then extrapolated to the remaining 23 appendicectomies for which operating time was also collected into the Excel spreadsheet from Bluespier, the operating theatre management system in use at our trust. The carbon footprint of the CO2 used was calculated based on a reference figure of 6 g CO2 per litre [20].

Electricity use

Electrical equipment pertains to all equipment directly used in the surgery that can be transported between different theatres and is plugged in for the duration of the surgery, unplugged when not used and stored away. This included the dithermy stack, the laparoscopic stack, pneumoperitoneum humidifier, Bair Hugger, second screen and smoke extractor. We calculated the energy use of equipment that fit these criteria by using plug-in wall energy meters (purchased from MECHEER EU from Amazon Marketplace). Energy use is known to be variable with plug-in equipment often based on the temperature the equipment is at and when the equipment is called to use (e.g., dithermy at the press of a button is activated), amongst other things. Hence, the energy stickers that note the wattage of the equipment cannot be used to accurately estimate the energy use of the equipment, as the usage varies throughout the operation. The plug-in meters were used for an appendicectomy and measured the total energy in kWh used by each of the equipment. The length of each appendicectomy was noted from patient entry into the theatre to closure; the equipment was on from the moment the patient entered the operating theatre and was turned off at closure. This reflects common practice at the trust from which the data was collected. The energy use per minute of operating time was calculated for all this equipment and extrapolated to all other appendicectomies using the data collected on operating time. Electricity usage was converted to kg CO2e using the reference figure of 0.155 kgCO2/kWh as per the HM's Department of Energy Security and Net Zero supplementary guidance to the Treasury Green Book. This figure is given in Table 1 for the year 2022, the last full year that data was collected for/revised; all later figures are modelled. It was advised in this documentation that footprinting is calculated using the grid average figure.

Heating, ventilation and air conditioning (HVAC) and overheads

Energy use in theatre overheads aims to estimate the energy use of the theatre with respect to heating, ventilation, and air conditioning (HVAC) and lighting. This was determined by using the information published for the 2022/2023 period in the National Estates Returns Information Collection dataset by site. This provided the energy use of the entire University Teaching Hospital of the Trust. The data was then averaged per square meter and per unit time (data was for the entire year; this was averaged per hour). This is a common method of calculating energy usage as per Morris et al. (The carbon footprint of cataract surgery) [15] and Connor et al. (The carbon footprint of a renal service in the United Kingdom) [17]. As per MacNeill et al., operating theatres are around 3-6 times more energy-intensive than the average of the entire hospital [10]; hence, the average for the hospital was multiplied by 4.5 to estimate the energy density of the operating theatre. The total area of this theatre was calculated, including the anaesthetic area, and multiplied by the energy density. This provided average energy usage for our theatre, and this was extrapolated for the entirety of operating time from patient entry into the theatre to closure.

Analysis

In order to analyse the data, it was first all transferred to an Excel spreadsheet. For each operation, the footprint of each mentioned category was calculated and then amalgamated. The range and mean were calculated for every category and the overall procedure. Then, the standard deviation (SD) and confidence intervals (CIs) were calculated for the procedure overall. Furthermore, the percentage of the total that each category made up on average was calculated, and equipment was further subdivided into reusable and non-reusable. Finally, Spearman's rank correlation coefficient was used to determine the correlation of operation time to variability in procedure footprint.

## Results

The mean carbon footprint of a laparoscopic appendicectomy was 28.3 kg CO₂e (SD: ±1.26), which can be divided into six categories of carbon sources, with the majority of emissions attributed to disposable equipment. The categories are pre-set equipment, variable-use equipment, scrub PPE equipment, pneumoperitoneum CO2 insufflation, equipment electricity usage and overhead (mainly HVAC) electricity usage. Pre-set equipment can be further broken down into reusable and non-reusable equipment, as opposed to variable-use equipment, which is always non-reusable. Of the PPE, only the gowns were reusable.

The average carbon footprint of each of these CO2 emission sources is given in Table 2. Briefly, the equipment made up 73.8% of the average footprint, of which reusable sources only contributed to 35% of this footprint. The most significant factor influencing the variation in carbon footprint was operation time, with a Spearman's rank correlation coefficient of 0.95. Pre-set equipment contributed 62.8% to the carbon footprint, whereas the use of equipment electricity only contributed 0.8% of the carbon footprint. The variation of the LA's carbon footprint was in the range of 24.4-36.3 kg.

**Table 2:** Carbon footprint of each equipment category PPE: personal protective equipment

Source of carbon	Footprint (gCO2)	Proportion of total footprint	Range (gCO2)
Pre-set equipment	17742.3	62.8%	0
Variable-use equipment	1356.3	4.8%	2510.3
Scrub PPE	1758.1	6.2%	986.2
Pneumoperitoneum	2578.0	9.1%	4136.1
Equipment electricity	228.1	0.8%	270.0
Overhead electricity	4595.8	16.3%	5439.4
Total	28258.6±1255.9	100%	11885.7

## Discussion

The LA is a carbon-costly procedure. CO2 emissions from LAs may be reduced by switching over to reusable equipment and increasing operating room efficiency. A good place to start for surgical teams is the green surgical checklist, which provides excellent recommendations, but surgical teams, with the help of this study, can go even further [22]. This study provided a brief overview of the surgical-side carbon footprint of a laparoscopic appendicectomy. It also outlined where the biggest usage of carbon is in this procedure, which can be used to guide footprint reduction measures. It also sheds light on where we are doing well in keeping footprints low, namely, the energy use of plug-in equipment; hence, we can direct efforts to working on other components. A large impact can be seen from reducing the footprint of the pre-set items and overhead electricity usage. Furthermore, more research is needed into less environmentally impactful gases to achieve a pneumoperitoneum: small gains can still have an impact cumulatively. It should be noted that the large range of pneumoperitoneum and HVAC footprints is grossly dependent on operative time, and as such, long operations overshadow the footprint of the increased equipment burden. There is hence a large case to be made for more efficient and experienced theatre teams saving carbon in the face of more complex cases.

There is limited data reporting other footprints of laparoscopic surgeries, which have often used an environmentally extended input-output model that relies on estimating the carbon footprint-based monetary cost of equipment. Alternatively, they utilise a process-based bottom-up approach, which looks at the process used for the creation of an item and converts this to a footprint using an established emission factor. A hybrid approach also exists using various degrees of both methods [11]. This study aimed to use a bottom-up approach using reference values published by Rizan et al. [20], with a majority of process-based methods. This, however, means it is susceptible to truncation errors based on the processes omitted by Rizan et al. It is not possible to determine the level of error ourselves, but we can be confident that there has been due diligence with regard to the equipment reference footprints, as it is peer-reviewed. Other studies have included pre- and post-operative factors such as travel, food and outpatient follow-up/medication [11]. With regard to the sample size, it is larger than many available sample sizes found in the literature. Rizan et al. used a sample size of 5-10 per operation to determine an inventory [20], whilst other papers have sample sizes of four [16] to 40 [15] for footprint calculations. Our method of calculating HVAC energy use was also previously used by others [15,17].

Overall, this study provides a robust estimate of the LA's footprint. The large sample size allowed us to be certain of the average length of an appendicectomy and the equipment used. The majority of the carbon footprint came from the equipment, which was accurately calculated using Rizan et al.'s data, and our estimates of the electrical equipment's energy usage were also very accurate using the plug-in wall meters. The only aspect of our estimate that may be less accurate is the HVAC theatre overhead estimate. There does not seem to be any accurate recording of the energy use of the average theatre at our site, and this varies between hospitals. Estate records were for a large site, not specifically the theatre itself, and so, estimates had to be extrapolated from this. This may make our results less generalisable as different operating theatres may vary widely in energy intensity as a result of size, HVAC systems and equipment density, amongst other things. Furthermore, different hospital sites will have different energy densities based on their infrastructure, making these results less generalisable. The use of a 4.5× multiplier to calculate the energy density of our theatres is another estimate that differs based on the site. It was not possible to determine where between three to six our trust lied, and so, a midpoint was used, but perhaps, a range may have been more generalisable.

The study also had other limitations, namely, we did not include other contributors to the footprint, such as travel for staff and patients, follow-up plans and post-operative recovery, nor the energy use of occupying a bed in a hospital in general. Whilst a large number of cases were collected, this does remain a single-centre study, and so, generalisability to other centres is limited. A multi-centre study could validate these findings. The study also did not incorporate the anaesthetic side of the operation, which comes with its own high footprint aspects, such as the induction gases used, lines and other tubes. Furthermore, the study can only assume that the overheads and equipment are turned off using only the energy average values for the site. It does not necessarily accurately reflect the footprint of when the HVAC system and equipment are left on, nor how often this is done. It could be the case that the emergency operating theatre is always left ready at sites; this would use a substantial amount of energy. All surgeons at the trust use a three-port technique initially; robotics are not used for this procedure, and all those converted to open techniques were excluded. This meant that the sample was representative for the technique. However, as the data shows, operative time was a major contributing factor to the footprint, and so, we assume difficulty. We believe that with our relatively large sample size, we have gained an acceptable case-mix of difficulty, but with larger sizes, one would gain a more representative one. An interesting point for future research could be how patient demographics impact the carbon footprint of the procedure, including age, body mass index (BMI) and comorbidities. Furthermore, collecting data on open appendicectomies would have been useful to compare the footprint of the techniques. This is, however, difficult as almost all appendicectomies are started closed, confounding the comparison. With regard to the pneumoperitoneum calculation, CO2 usage can vary based on the pre-set pressure settings and surgical technique (e.g., resulting in an air leak); these were not accounted for. As always with carbon footprinting, you can only be as accurate as the factors you include. Undoubtedly, there will be aspects of the footprint that will have been missed and make this likely an underestimate.

A few items were not included in the estimate, such as the laparoscopic staplers or clips. These items can have a significant impact on the footprint, with some research estimating the footprint of the clip applicator being 2559 gCO2e [23] and an electric stapler being 8.54 kgCO2e or a manual endoscopic stapler being 2.97 kgCO2e [24]. No data is available on the footprint of Veress needles or Robinson drains, the only other equipment not included in the calculations. Assuming our staplers were manual, with both clips and staplers being used twice each, the average appendicectomy would have been calculated at around 480g CO2e more, around a 1.7% increase. This is not insignificant and shows the importance of more research being needed into the footprint of all equipment used. This is not included in our estimate as we are unable to accurately determine the exact product used in the literature.

Some work has already been put into addressing the footprint of appendicectomy by Labib et al. as part of a Sustainable Quality Improvement (SUSQI) project on improving the pre-set instrument list in an audit of the equipment used. This created a more efficient laparoscopic set for appendicectomy based upon what single-use equipment was opened [18]. In our work, the variable-use equipment made up a relatively small proportion of the footprint (~4.8%), suggesting that our trust has a good grasp on which equipment is required. However, the equipment used in the 'pre-set' items is often single-use, such as the ports, drapes and scissors. 

## Conclusions

The implications of this study for making the operating room more energy-efficient are broad. The study has proved that if the operation becomes more efficient, it also becomes greener, if this does not require the use of any more equipment. A well-trained, efficient theatre team with surgeons who are experienced can save carbon. It has also been shown that equipment has the lion's share of the footprint and that changes to the footprint of the equipment have far-reaching implications. One such option could be to revert to the use of multiple-use ports rather than disposable ones. Carbon dioxide has been the insufflation gas of choice to create a pneumoperitoneum for several years, but this inert gas is the culprit in climate change. More research is needed into whether other inert gases with a lower footprint can be safely used to create a pneumoperitoneum. By incorporating all these recommendations and embracing the principle of marginal gains when reducing the footprint in multiple small ways, theatre teams can have a significant impact on the sustainability of the operating room. Even the reusable items have been shown to have a large footprint. If the operating theatre is to have a chance at reaching net zero by the NHS's target, the NHS must start to offset its carbon emissions. It is impossible to design a zero-carbon procedure without this. To conclude, this study provides granular data to guide targeted interventions and quantify the degree of offsetting required to achieve net-zero emissions in laparoscopic surgery.

## Figures and Tables

**Figure 1:**
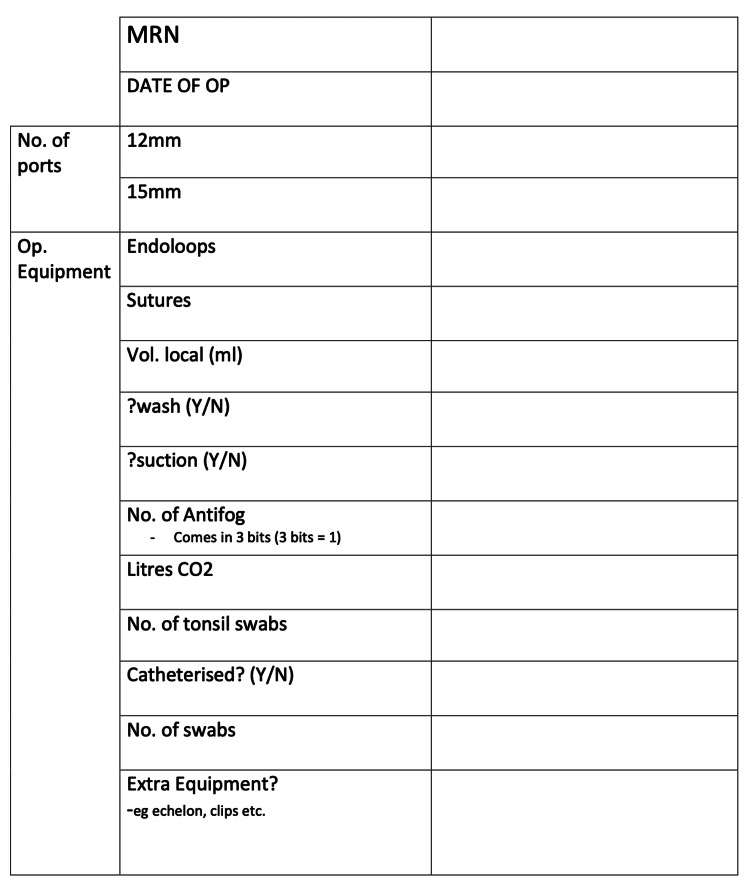
Data collection sheet

## Appendices

The data collection sheet used by the surgical team is presented in Figure 1. 
